# Effects of Curcumin and Sulforaphane on C. elegans Healthspan: Independent and Combined Impacts

**DOI:** 10.1093/geroni/igaf122.2541

**Published:** 2025-12-31

**Authors:** R P Vivek-Ananth, Stephen Phipps, David Weinkove, Nathan Price

**Affiliations:** Buck Institute for Research on Aging, Novato, California, United States; Thorne HealthTech Inc., New York, New York, United States; Durham University, Durham, England, United Kingdom; The Buck Institute, Navato, California, United States

## Abstract

Aging involves a progressive decline in bodily functions, underscoring the need for interventions that enhance healthspan—the period of life spent in good health. Natural products like curcumin and sulforaphane show promise in modulating aging-related pathways; however, their combined effects remain largely unexplored. This study evaluated the healthspan-enhancing potential of nine natural products in Caenorhabditis elegans, focusing on the individual and combined effects of curcumin and sulforaphane. Using high-throughput automated phenotyping with WormGazer™, we measured movement-based healthspan metrics—the fraction of moving worms, mean speed, and distance moved—from days 2 to 7 of adulthood. Toxicological evaluations confirmed that all compounds were non-toxic at the selected concentrations. Curcumin increased the fraction of moving worms in early and late adulthood, although its positive effect on speed was limited. In contrast, sulforaphane enhanced speed and distance moved in late adulthood but had variable effects on early mobility. Notably, the combination of curcumin and sulforaphane produced effects similar to those of sulforaphane alone, with no additive benefits observed across mobility metrics. Transcriptomic analyses revealed distinct gene expression profiles for each treatment. Further AI-based analysis identified divergent molecular targets and pathways, explaining the differing effects of the compounds. These findings highlight the complementary roles of curcumin and sulforaphane in improving healthspan metrics in C. elegans. While both compounds independently enhance aspects of mobility, their lack of synergy underscores the complexity of multi-target interventions in aging biology. This study offers new insights into the molecular mechanisms modulated by natural products and supports their potential in promoting healthy aging.

